# Development of an integrated, holistic care package for people with lymphoedema for use at the level of the Primary Health Care Unit in Ethiopia

**DOI:** 10.1371/journal.pntd.0009332

**Published:** 2021-04-20

**Authors:** Abraham Tesfaye, Maya Semrau, Oumer Ali, Mersha Kinfe, Mossie Tamiru, Abebaw Fekadu, Gail Davey

**Affiliations:** 1 Center for Innovative Drug Development and Therapeutic Trials for Africa (CDT-Africa), Addis Ababa University, Addis Ababa, Ethiopia; 2 Brighton and Sussex Centre for Global Health Research, Brighton and Sussex Medical School, University of Sussex, Brighton, United Kingdom; 3 Ethiopian Ministry of Health, Addis Ababa, Ethiopia; 4 School of Public Health, Addis Ababa University, Addis Ababa, Ethiopia; Federation University Australia, AUSTRALIA

## Abstract

**Background:**

Neglected Tropical Diseases (NTDs) are a group of several communicable and non-communicable diseases prevalent in tropical and subtropical areas. The co-endemicity of these diseases, the similarity of their clinical signs, and the need to maximize limited financial and human resources suggest the importance of adoptingan integratedapproach to their prevention and treatment.

**Aims:**

This study describes the development of a comprehensive package of physical, mental health and psychosocial care for people with lower-limb lymphoedema caused bypodoconiosis, lymphatic filariasis (LF)or leprosy as part of the EnDPoINT program in Ethiopia.

**Method:**

The care package was developed using a mixed-methods approach, consisting of a literature review, situational analysis, Theory of Change (ToC) workshops, qualitative research, and additional workshops to fine-tune the draft care package. The care package was developed between March 2018 and January 2020 in Addis Ababa and the implementation research site, Awi zone in the North-West of Ethiopia.

**Results:**

The holistic care package includes components implemented at three levels of the health care system:health organization, facility, and community. Sections of the care package are directed at strengthening capacity building, program management, community engagement, awareness-raising, stigma-reduction, morbidity management, disability prevention, follow-up visits, referral linkage, community-based rehabilitation, and monitoring and evaluation.

**Conclusions:**

The study developed a holistic integrated care package for lower limb disorder and co-morbid mental health problems caused by podoconiosis, LF or leprosy. The approach has the potential to significantly reduce lower limb disorder-associated morbidity, disability, and psychosocial problems. It also standardizes a scalable approach appropriate for the Ethiopian setting and, most likely, other countries where these NTDs are present.

## Author summary

Neglected tropical diseases resulting in lower limb lymphoedema (localized swelling by an accumulation of lymph) such as podoconiosis, lymphatic filariasis (LF) and leprosy are common in Ethiopia. Knowledge of the care management of lymphoedema of these neglected diseases within health care systems is often inadequate. Similarly, the mental distress and illness that commonly accompanies lymphoedema often go untreated. This study developed an integrated holistic care package for people with lymphoedema. The care package involves physical, mental and psychosocial interventions for patients with podoconiosis, LF and leprosy for use at the health care administration, health facility and community level in Ethiopia. The findings of this study inform plans for implementation of integrated foot care and psychosocial support interventions across Ethiopia and beyond.

## Introduction

Lower limb lymphoedema isthe common manifestation of a group of communicable and non-communicable diseases. Podoconiosis, lymphatic filariasis (LF) and leprosy are amongthe ‘skin’neglected tropical diseases (NTDs) that cause lower limb swelling and are common in tropical and subtropical areas amongpoor and remote communities with low access to the health system.Podoconiosis is a form of non-filarial elephantiasis caused by prolonged barefoot exposure to irritant red clay soil on a background of genetic susceptibility[1]. Lymphatic filariasis (LF) isa parasitic disease caused by*filarial* worms and leads to swelling of the legs, arms and genitalia[2]. Leprosy is a chronic infectious disease caused by *Mycobacterium leprae*[3].

The global burden of lymphoedema attributable to LF, podoconiosis, and leprosyis estimated to be 16 million[4], 4 million[5], and 200,000[6], respectively. These conditions are common in Ethiopia, where podoconiosis, leprosy,and LF account for approximately 64.8% [7], 12.8%[8], and 0.3% [9]of the total burden of lymphoedema in Ethiopia respectively. In 2015, burden assessment in LF-podoconiosis co-endemic districts demonstrated up to 2,227 patients with lymphoedema in a district of approximately 100,000 inhabitants [10]. These diseasescan have a significant impact onaffected individuals and communities in terms of disability, mental distress, depression, stigma, and loss of economic productivity (10,11). The estimated economic burden of podoconiosis in Ethiopia’s economy is US$213.2 million annually and 91.1% of this results from loss of productivity [11].

The integration of NTDs into the activities of the primary health care unitand further integration of control programs among co-endemic NTDs has been a priority in global health. Integration could advance the control of disease across a range of domains, including mapping, diagnosis, clinical management, and community control measures such as mass drug administration.The Ethiopian Ministry of Health has recognized the importance of integration, and as a result, both podoconiosis and LF have now been included in the first two National Master Plans (2013–2015 and 2015/16-2019/20), and in 2016, a joint morbidity management and disability prevention guideline was developed.However, this did not include leprosy, mental health services, or psychosocial support[12,13].

In Ethiopia, foot care for leprosy patients has been integrated into routine health services [14], while foot care interventions for podoconiosis and LF are currently mainly provided through donor-supported projects in a disease-specific and disparate manner. Knowledge of these neglected diseases within health care systems is therefore often inadequate, and diagnosis and treatment options are limited.Besides, the mental distress and illness that commonly accompany lymphoedema [10,15,16] often go untreated [17], and mental health services are provided by higher-level health facilities rather than the level of the primary health care unit. There is a clear rationale for the integration of care across these conditions into the primary health care unit because of the shared clinical symptoms of lower-limblymphoedema,their co-endemicity,the current lack of provision by government health centers (for LF and podoconiosis) and the frequency with which these diseases are accompanied by mental health problems.

The main objective of this study was, therefore, to develop a holisticintegrated care package for lower-limb lymphoedema caused by podoconiosis, LF, and leprosy, which included mental health and psychosocial care alongside physical services. The study’s primary research question was: what are the key elements that constitute optimal physical and psychosocial care for patients with podoconiosis, LF and leprosy? The packagewill helpstandardize care for patients with lower-limb lymphoedema and integrate this into government-run health services. The study was part of the ‘Excellence in Disability Prevention Integrated across NTDs’ (EnDPoINT) program[18].

## Methods

### Ethics statement

*Ethical approval for the study was obtained from the BSMS Research Governance and Ethics Committee (ER/BSMS9D79/1 and ER/BSMS9D79/2) in the UK, as well as the Institutional Review Board of the College of Health Sciences of AAU (061/18/CDT) in Ethiopia. Formal written consent was obtained from all participants*.

The care package was developed using a mixed-methods approach, consisting of a literature review, situational analysis, Theory of Change (ToC) workshops, qualitative studies, and additional workshops to fine-tune the draft care package. The care package was developed between March 2018 and January 2020 in Addis Ababa and the implementation research site, Awi zone in the North-West of Ethiopia.

### Study setting

Awi zone is one of 10 zones in the Amhara regional state of Ethiopia, situated between 11° 00’ 0.00" N and 36° 39’ 59.99" E, and about 469 km north of the capital city, Addis Ababa. The total population of the zone is 1,207,908 people (female 603,563 and male 604,345) with about 82.2% living in rural areas in 2017[19]. The zone is divided into 3 urban and 9 rural districts and covers a geographic area of 9,148 square kilometers. The elevation varies from 1,800 to 3,100 meters above sea level, with an average altitude of about 2,300 m. There are three main climate types: ‘*Kola*’ (tropical), ‘*Woina dega*’ (sub-tropical), and ‘*Dega*’ (cool). Awi zone was selected as the study site because of the established co-endemicity of podoconiosis, LF, and leprosy and because it represented the climatic diversity found within Ethiopia.

### Literature review

A document review was conducted as a first step towards informing and guiding the development of the holistic care package. The national NTD Integrated Morbidity Management Guideline [20], National Neglected Tropical Disease Master Plan [21], the National Mental Health Strategy[22], the WHO Mental Health Gap Action Programme (mhGAP) Intervention Guide[23], the Ethiopian Health Sector Development Plan[24], the Ethiopian Demographic and Health Survey[25], relevant program documents and relevant publications in scientific journals were reviewed. Lymphoedema, podoconiosis, lymphatic filariasis, leprosy, mental health, and care package search terms were used.

### Situational analysis and resource assessment

To understand the local context for the development and implementation of the holistic care package, a cross-sectional situational and resource assessment was carried out using a situational analysis and resource assessment tool (downloadable from thelink below). The tool was adapted from previous situational analysis and asset mapping tools used in another implementation science project, the ‘Programme for improving mental health care’ (PRIME) project (see http://www.prime.uct.ac.za/situational-analysis-tool). The information was collected through desk reviews and interviews with purposively sampled key officials (district, zone, and region level health and planning officesheads) and service managers.

The situational analysis tool consisted of four sections: relevant context, health services, podoconiosis-NTD coordination and planning, and community. The ‘relevant context’ section included 44 questions on socio-demographics, economics, general health, NTDs, lymphoedema management, human immunodeficiency virus (HIV)/tuberculosis(TB), mental health, and policies and strategies. The ‘health services’ section included 77 items on health facilities, human resources, mental health services, surgical care, medical equipment, and medication. The ‘podoconiosis-NTD coordination and planning’ section included 40 items on zone administrative structure, political support, financing, NTD program plan, coordination, human resource planning, health information, technologies for lymphoedema health care, service organization and transmission control. Finally, the ‘community’ section included 19 items on socio-cultural factors, non-health sector activities, health promotion, health professionals and volunteers, patient associations, and key stakeholders.

### Theory of change workshops

‘Theory of Change’ (ToC) has been defined as a “theory of how and why an initiative works” [26]. It is a participatory structured thinking process to program design and evaluation. It helps to build a causal pathway of what needs to change and why. It provides a clear explanation of the mechanisms of change through which the intervention leads to real-world impact. The information gathered through ToC workshopsis essential in developing complex interventions that are more likely to be effective, sustainable, and scalable.

Three workshops were conducted to map out the care package’s ‘Theory of Change’ (ToC) in terms of defining its desired outcomes, indicators, interventions, and outcome measurements, and to encourage stakeholder buy-in to the study.

Each of the ToC workshops was started by introducing the participants, an overview of the upcoming activities of the day, and a welcoming note. The introduction session was followed by an overview of theEnDPoINT project and an introduction to the Theory of Change. Finally, the participants engaged in the process of building the ToC framework. Building the framework was started with defining the intended impact, then by working backward to determine the intermediate and short-term outcomes necessary and sufficient to achieve the intended impact. Indicators for each outcome were identified, the rationaleof how one outcome leads to the next was articulated, and interventionsrequired to achieve the outcomes were identified and agreed amongst stakeholders. Finally, the ToC was displayed as a ToC map that illustrates the ToC process and provides a framework for evaluation (**S1 Appendix**). The details of the methods are described elsewhere[27].

A first draft of the ToC map was developed on May 16 and 17, 2018, by 20 individuals who were members of the EnDPoINTConsortium and research team. The participants’ organizations were universities (Addis Ababa University, Brighton and Sussex Medical School (BSMS) and Imperial College, London), the Ethiopian Ministry of Health, and non-governmental organizations working on podoconiosis, leprosy and LF (International Orthodox Christian Charities, Ethiopian National Association of Persons Affected by Leprosy[ENAPAL], and National Podoconiosis Action Network[NaPAN]).

The ToC map was further expanded and refined by 34 individuals during the second workshop on October 12, 2018. The participants were from the project implementation region, zone, district (‘woreda’), health facility, or community level. The workshop attendee profile included the regional health bureau disease prevention department head, zonal & district health office heads, district heads, health center heads, NTD team leaders, researchers at the regional public health institute, health professionals (related to podoconiosis, LF, leprosy, and mental health), patients (podoconiosis and LF), religious leaders, community leaders, community health workers, and district level education, women affairs and small scale enterprise office heads. Non-governmental organizations working in the area also participated in the workshop. The workshop was conducted in the local language (Amharic) in Bahr Dar city, close to the project implementation districts.

The ToC map was finalized by 20 individuals during the third workshop on December 17 and 18, 2018. These were some of the participants who had earlier participated in the first or second workshops. The first and third workshops were conducted in English.

### Qualitative research

Three focus group discussions (community, health professionals, and policymakers), each consisting of 7–10 participants, and key informant interviewswith 11 participants were conductedto test thefeasibility, acceptability, and appropriateness of the draft care package in terms of its integration into government-run health services. The community and Health professional focus group discussions were held in the project implementation site (Awi zone). The policymakers’ focus group discussion was held in Bahrdar city and attended by policymakers from Amhara regional state. Key informants were from the region and national offices. The detailed methods and results of this part of the EnDPoINT project will be submitted as a separate manuscript.

### Workshops to discuss draft care package

Two workshops were held to discuss and fine-tune the draft care package and accompanying training materials. The first workshop was held in the capital city, Addis Ababa, and attendees were from the Ministry of Health, the project members and stakeholders at the national level. The focus of the workshop was to get feedback on the documents from a national and international perspective. The second workshop was attended by health professionals and stakeholders from the project implementation site. The workshop aimed to get comments from the local context.

### Piloting

The care package was piloted in the Gusha health center and its surrounding catchment area community in Awi zone.The main climate type of the area is ‘*Woina dega*’ (sub-tropical) and the area was selected due to the presence of the three cases (LF, podoconiosis, and leprosy) and recommendation by the zonal health bureau. Following the pilot study and subsequent workshopon the result of the pilot study, the care package was finalized and ready for scale up to three districts in Awi zone. The detailed methods and results of this part of the EnDPoINT project will be submitted as a separate manuscript.

## Results

The holistic care package includes components implemented at the level of the health care organization, the health facility, and the community (Tables 1–3). Sections of the care package are directed at strengthening capacity building, program management, community engagement, awareness-raising, stigma-reduction, morbidity management and disability prevention (MMDP), follow-up visits, referral linkage, community-based rehabilitation, and monitoring and evaluation.

**Table 1:** Health Organization (HSO) level interventions.

No	Intervention	Objective	Activity	Evaluation
1	Awareness-raising and mobilization workshop	To raise awareness about: Lower limb care and co-morbid mental disorders and their treatability; Raise understanding of the broader public health and development benefits of intervention through the treatment of lower limb and co-morbid mental disorders.	Half-day workshop	Qualitative evaluation of change in attitudes and awareness over time
2	Program management support	To ensure proper management of the program including planning, dedicated budget, regular availability of supplies, trained health workers, proper coordination and monitoring & evaluation.	Working alongside and in partnership with key personnel in the health service organization (HSO)throughout project implementation	Qualitative evaluation on the effectiveness and efficiency of the program
3	Training of Trainers (ToT) on the lower limb and co-morbid mental health care	To improve the capacity of health workers on podoconiosis, LF, leprosy, and co-morbid mental health disorders	Six days of training	Pre-post-test and teach-back evaluation, attendance
4	Training of Trainers (ToT) on supportive supervision, mentoring and coaching	To be able to provide supportive supervision to primary health care unit (PHC) workers to monitor the quality of care	Two days of training	Pre-post-test and teach-back evaluation, attendance

**Table 2:** Health Facility Interventions.

No	Intervention	Objective	Activity	Evaluation
1	Provider MMDP training	To enable primary health care staff to provide competent care to patients with lower limb disorder caused by podoconiosis, lymphatic filariasis and leprosy, and co-morbid mental health problems	Five days (3 days MMDP and 2 days mental health) of theory training and 5 days of practical training on mental health	Pre-post-test
2	Provider supply chain management training	Improve understanding of basic supply system concept of Ethiopian Pharmaceutical Fund and Supply Agency; Improve knowledge to acquire stock, prevent stock-out, and manage supplies.	One day training	Pre-post-test
3	Awareness-raising workshop for health facility staff	To improve awareness about the diseases and MMDP benefits; To reduce stigmatizing attitudes; To raise awareness about the benefits of providing inclusive care.	Half-day participatory workshop	Number of attendees
4	Awareness-raising workshop for general attendees at the health center	Learn about symptoms, causes, prevention, and treatability of lower limb disorder and co-morbid mental disorders; Reduce stigmatizing attitudes and discriminatory practices.	30 minutes per week of health education; Poster in waiting rooms	Number of health education sessions per month; Number of health centers with posters in place
5	Assessment, diagnosis and treatment initiation	To provide comprehensive and holistic care for patients with lower limb disorder and co-morbid mental health disorder	Patient training in self-care (hygiene, skincare, elevation and exercise, footwear, wound care) at the health center; Patient counseling and co-morbid mental health care; Provide MMDP supplies; Referral linkage	Clinical and psychosocial patient outcome improvement; Number of patients who received MMDP care and supplies

**Table 3:** Community Interventions.

No	Intervention	Objective	Activity	Evaluation
1	Community health worker MMDP training	To enable health extension workers to support patients with lower limb disorder and co-morbid mental health problems	Two days of training	Pre-post-test
2	Community conversation (CC) facilitator training	To create a competent facilitator of community conversation	Threedays of training	Pre-post-test
3	Self-help group facilitator training	Two create competent facilitator of self-help group	Two days of training	Pre-post-test
4	Awareness-raising workshop	To increase awareness of the disease and wearing shoes; To reduce stigma and discrimination; To increase service utilization; To facilitate social reintegration	A workshop in local vicinity including testimonials from patients/caregivers	Number of CC meetings; Change in community knowledge, attitudes, and practice; Availability of posters and billboards
5	Community Conversations		Twice a month regular CC meeting	
6	Information dissemination		Information dissemination through leaflets, posters, billboards and mass media	
7	Community-based case finding and referral	To identify and refer cases to the health center	Regular case finding through community health system network	Number of cases linked to the health center
8	Follow-up visit	To improve adherence and quality of home-based self-care routine; Monitoring of mental state, detecting early signs of acute attacks and referring for review when needed	Visiting patients at their home by community health workers	Number of visits and referrals
9	Socio-economic rehabilitation	To empower people with disabilities; To engage the family, community, and relevant organizations in providing opportunities and support for the disabled.	Establish patient self-help groups and patient associations; Meetings with community leaders and stakeholders	Access to rehabilitation services and increased productivity
10	Supportive-supervision (a cross-cutting issue for the health organization, health facility, and community levels intervention)	To ensure the practice is in line with the plan; To identify and overcome short-comings	Quarterly on-site supportive supervision	Supportive supervision feedback reports

### Capacity building training

Health center-level provision of quality services for lower limb disorders and co-morbid mental health issues is necessary both to manage the conditions and to prevent further medical, psychological, economic, and social complications. The provision of quality services is in part achieved by providing essential knowledge and skills to health professionals. The care package, therefore, encompasses a range of capacity-building activities for trainers and end-user training on podoconiosis, LF, leprosy, mental health, supply chain management, supportive supervision, mentoring, and coaching. Training of Trainers (ToT) is necessary to build capacity within the district to be able to deliver training to new staff members, thereby enhancing institutional culture, facilitating continued learning, and ensuring the sustainability of the service. Initially, the mental health training for primary health care workers was planned for two days (as part of 5 days MMDP training) but based on feedback at the start of piloting; it wasimmediately adjusted to an additional 5 days of practical training on mental health. The providers were trained on the theory of self-harm/suicide, depression, psychosis, and alcohol/substance-use managementusing the WHO Mental Health GAP Action Programme (mhGAP) training manual. During the practical session, the providers were trainedon diagnosis, case management, and psychosocial support ofthemental health cases at hospital-level bysenior psychiatrists.

### Program management

Besides improving the competency of health professionals, ensuring regular availability of supplies for MMDP for lower limb disorders and co-morbid mental health care is required. The supplies required to treat patients have been listed in the care package, and some items have been suggested for free-of-charge distribution. To ensure the sustainability of the supplies, the care package enables the inclusion of these items into regular health product distribution by the Ethiopian Pharmaceuticals Supply Agency. The package also facilitates the issuing of ‘free certificates’ to those who are disabled so that they can access free health care through the Health Insurance scheme.

### Community engagement

To facilitate the engagement of the community in program implementation, the care package suggested the establishment of a Community Advisory Group. The group was composed of representatives from the district administration, health office, education office, labor and social affairs, credit and saving institute, religious leaders, patient associations, and women, youth and children affairs.

### Awareness raising and stigma-reduction

During the ToC workshops, we learned that stigmatization and discrimination of people withlower-limb disorder and their family members are common. The situation is pronounced and results in the exclusion of patients from school, social, and religious functions. Patients and unaffected family members are commonly excluded from marriage. Society attributes the three diseases to evil spirits, and it is assumed that the spirit runs in the family, causing the conditions. This perceived cause of the illness affects the treatment modalities that the patients seek. To counter the discrimination against affected people, the care package includes awareness-raising activities (awareness-raising among general attendees at the health center, community awareness-raising workshops, community conversations, and information dissemination[lefleats, billboard, and posters], the establishment of a self-help group and patient association, income-generating activities, active case finding and referral by community health workers, patient home follow-up visits and subordinate interventions). Visiting patients at their home also helps to detect clinical complications early, remind patients and their families of the basic management techniques, improve adherence, monitor their mental state, and refer or review when needed. Community conversation facilitator training for 3 days was included in the package to conduct a purposeful and meaningful community conversation. The purpose of the community conversation is to bring together members of the community and encourage them to discuss and explore the main causes and underlying issues behind lymphoedema and solve the problem.

### Morbidity management and disability prevention

Providing comprehensive and holistic care for patients with lower limb disorders and co-morbid mental health problems is vital for improving patient quality of life and productivity. The care package encompasses case finding, assessment, diagnosis, treatment services, patient counseling, and co-morbid mental health care to be implemented in the primary health care unit. It includes patient training in self-care: hygiene, skincare, elevation and exercise, footwear, and wound care. The patients were treated as outpatients and referred if further care was required.

### Case finding

Health extension workers are community health workers with one year’s training who are assigned to local health posts. They are ideally placed to improve case detection in the community. This approach to case detection is recommended in the care package and fits closely with their expected roles and responsibilities for other disorders. Once established as part of their role, this has the potential to bring about sustainable improvements in case finding and referral linkage. To reduce the burden on HEW, the community health system involves community volunteers known as the ‘Health Development Army’ (HDA). These volunteers (primarily women) are supposed to promote the uptake of key programs by mobilizing the population and supporting the HEWs. They are representatives of their communities. The care package recommends the use of HDA in addition to HEW in case finding and implementation of the interventions.

### Follow-up visits

Visiting patients at their home helps for early detection of entry lesions to prevent secondary bacterial infections, remind patients and their families about basic management techniques, and ensure continuation with self-care.

### Community-based rehabilitation

The potential of people with disabilities from lower limb and co-morbid mental health problems is frequently overlooked and as a result, they are often excluded from income-generating, education, and employment opportunities. This part of the care package aims to help people with disabilities, by establishing community-based programs for physical rehabilitation, social integration, and equalization of opportunities. Self-help group facilitators were selected from the health development army and trained for two days. The self-help groups composed of people with lymphoedema were established at the village level. The patient association was established at the district level.

### Monitoring & evaluation

Supportive supervision is thought to be important for the sustainable integration of health interventions into the Primary Health Care Unit (PHC). The health system has a supervisory framework for reaching the PHC workers, but supervisors may not have an adequate background in lower limb disorder or co-morbid mental health care. This part aims to strengthen the provision of supportive supervision to PHC workers to monitor the quality of care, including the development of the holistic care supervision checklist.

## Discussion

Based on a request from the Ethiopian Ministry of Health, this paper describes the development of a scalable integrated holistic care package for lower limb disorderand co-morbid mental health problems caused by podoconiosis, lymphatic filariasis(LF), or leprosy. The holistic care package includesinterventions to be implemented at the level of the healthcare organization, health facility, and the community. The package has the potential to assist health professionals, program managers, and development partners with program design and implementation. The interventions have the potential to help with capacity building, community engagement, morbidity management, disability prevention, and alleviating mental health and socioeconomic problems.

The care package is designed according to the multidimensional needs of patients, health professionals and the community, and is managed by a coordinated multidisciplinary team of individuals working across settings and levels. This team includes community members (patients, religious leaders, elders, and local administration officers) to ensure optimal outcomes and the appropriate use of available resources. The interventions were systematically developed using a literature review, situational analysis, Theory of Change (ToC) workshops, qualitative research, and workshops to discuss the draft care package. The community’s participation in both outlining the problems and devising appropriatesolutions was notable. During the second ToC, the depth of stigma and discrimination in the community was conveyed in terms ofhaving an enormous impact on stakeholders. The diverse combination of the participants during the ToC workshops helped to generate honest discussion and solutions from different perspectives. Separating the first and second workshops based on education level and language (Amharic local language and English) was important to avoid languageand power-related barriers.

The care package is in alignment with the WHO global strategy on people-centered and integrated health services[28]. The WHO recommends the implementation of integrated control interventions that take into account all co-endemic NTDs in a given community. The care package creates connectivity, alignment, and collaboration within and between organizations, health facilities, and communities. It makes specific contributions by integrating mental health services into lower limb disorder morbidity management. Integrating the services into primary health care units is expected to render the servicesmore accessible to all individuals and families in needwithin the community. The care package promotes the comprehensive delivery of quality services across the life-course at the primary health care unitand hasthe potential to improve the quality of life of patients.

The mental health services were planned to be implemented by primary health care health professionals (not trained as psychiatrists) due to a shortage of psychiatrists. There are few psychiatrists with a ratio of~1.5 psychiatrists to 2 million people in Ethiopia [29]. Initially, the mental health training was planned for two days (as part of 5days MMDP training), but during pilot, we learned that additional practical training was a necessity to provide the service at the piloting health facility. We adjusted the MMDP training duration to 10 days including a5-day practical session on mental healthand added in-service mentoring by a psychiatrist one day per week for three months. A study in Uganda reported that one key health system constraint to integrating mental health services into PHC was inadequate practical experience during training[30]. In the study, at the beginning of the pilot phase, thePHC health professional participants indicated that they did not feel confident to handle mental health problems and needed additional training. For scale-up of the care package, we adjustedthe mental health training to 10 days including 5days of practical sessions and facility mentorship support by a psychiatrist. The WHO has recommended the implementation of mental health services at the primary health care unit and it is a central idea to the values and principles of the Alma Ata Declaration[31].

Rigorous evaluation of the components of the care packages is ongoing in three districts in Awi zone. The findings (including outcome and cost-related issues of the interventions) will be shared in another paper. Surgical issues like hydrocele and some rehabilitation services such as leg rehabilitation for leprosy patients are not within the scope of the care package. Community Health workers are more often engaged in societal beliefs about disease causation. Therefore directly engaging community health workers in interventions that engage with the perceived spiritual causation of disease might be a threat to the care package and one of the limitations of the paper.

## Conclusion

This study describes the development of a holistic integrated care package for lower limb disorder and co-morbid mental health problems caused by podoconiosis, lymphatic filariasis orleprosy. The approach has the potential to significantly reduce lower limb disorder-associated morbidity, disability, and psychosocial problems.

## Supporting information

S1 Appendix ToC Map. (PDF)
