# Supplementary material for: Development of an integrated, holistic care package for people with lymphoedema for use at the level of the Primary Health Care Unit in Ethiopia
Source: PLoS Negl Trop Dis. 2021 Apr 20;15(4):e0009332. doi: 10.1371/journal.pntd.0009332 (PMC8086999; doi:10.1371/journal.pntd.0009332)
Supplement: S1 Appendix — (PDF) [file pntd.0009332.s001.pdf]

Supporting information: 'Theory of Change' Map

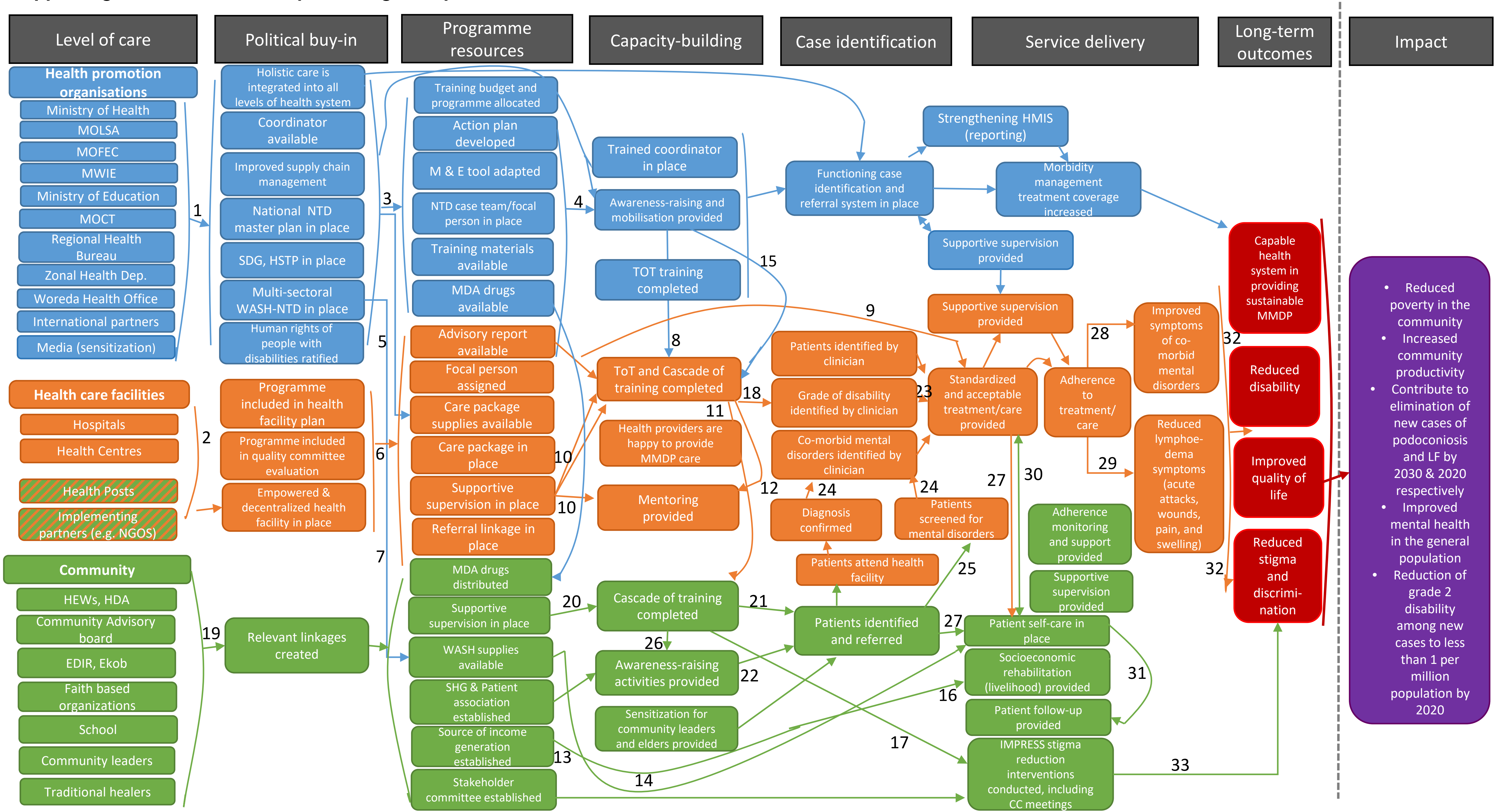

Assumptions

1a Willingness of stakeholders at health promotion organisation level to participate/engage
2 Willingness of health facility staff to participate/engage
3a Adequate funding available
3b Capacity/resources available to develop action plan
3c Adequate routine data collection
3d Capacity/resources available to adapt M & E tool
4 Availability/willingness of stakeholders to participate in capacity-building activities
5 Adequate structures in place for medical supplies to reach health facilities
6a Possible to make advisory report available
6b Availability of supervisors
7 Organisations such as WASH are willing to collaborate
12 Willingness/engagement of HEWs to participate in training
13 Willingness of stakeholders and patients to engage/participate
14 Structures are in place for WASH supplies to reach patients
15 Health facility staff are willing and available to participate in capacity-building activities
16 Income generation sources/activities sustainable
17 Willingness of community to participate in stigma reduction activities
18/21/22 Training is adequate
19 No opposition from community, e.g. FBO, schools or traditional healers
23a Staff are able to provide care, e.g. sufficient capacity, experience, resources etc.
23b Trained staff remain in post and new staff are trained
24 Community members willing and able to visit health facilities, and to receive treatment
25a Screening mechanisms are adequate
25b Patients consent to being assessed/screened for mental health
26 Willingness of community to participate in awareness-raising activities
27 Patients able to perform self-care, i.e. sufficient capacity
28 Adequate treatment leads to improved mental health outcomes
29 Adequate treatment leads to a reduction in lymphoedema symptoms
30 Patient self-care is adequate
31 Patients consent to follow-up
32a Improved mental health outcomes result in the long-term outcomes
32b Reduced lymphoedema symptoms result in the long-term outcomes
33 Stigma reduction interventions lead to reduced stigma

Interventions

Health promotion organization level

1 Programme management at zonal and district level
4a Conduct participatory sensitization workshops
4b Convene public advisory group
5 Advocate/lobby for medical supplies to be made available in health facilities
7 Ensure that WASH supplies are made available
8a Capacity-building in care provision at health care organisation level (ToT)
8b Capacity-building in supervision at health care organisation level (ToT)

Health care facility level

9a Supply chain management
9b Provide training on supply change management
10a Capacity-building of health facility staff in podoconiosis, LF and leprosy
10b On-site supportive supervision for health facility staff
11 Clinical mentoring for health facility staff
15a Awareness-raising and sensitization workshops for health facility staff
15b Posters in health facilities
15c Health education sessions for attendees of health facilities
18 Case detection by health facility staff
23a Assessment, treatment and care initiation by health facility staff
23b Institutional-based rehabilitation (minor surgery; links with orthopedics, physiotherapy; rehabilitative surgery; shoe makers)
27 Training of patients in self-care

Community level

12 Capacity-building of HEWs and CC facilitators
14 WASH supplies delivered to patients
16 Community-level socio-economic rehabilitation
17a Conduct community conversation (CC) sessions
17b Patient self-help and/or peer support groups
17c Provision of health information by HEWs
17d Educational media campaign
17e Coping skills acquisition / counselling for patients
19 Engage with and sensitise key stakeholders at the community level
20 On-site supportive supervision for HEWs and CC facilitators
21 Active case detection by HEWs
22 Patient self-identification
24 Referrals by HEWs to health facilities
25 Mental health screening by HEWs
26 Conduct awareness-raising sensitization workshops
31 Follow-up home visits by HEWs

Indicators

Health promotion organization level

1 Implementation of programme; MoU signed if necessary; number of meetings with officials
4a Improved attitudes and awareness (qualitative evaluation)
4b Number of times group meets; all key stakeholders included in group
5 Number of health facilities to which medical supplies are made available
7 Number of WASH supplies distributed
8a Improved ability to train others others (pre-post test and teach back evaluation)
8b Improved supervision skills

Health care facility level

9a Number of stock-out days for main supplies
9b Improved KAP post-training compared to pre-training
10a Competence improved post-training compared to pre-training; knowledge, attitude and motivation improved; training activities satisfactory
10b Number of supervision sessions
11 Number of mentoring visits conducted; number/types of skill gaps filled
15a Improved behaviour towards patients (measured through qualitative patient interviews)
15b Number of health facilities with posters in place
15c Number of times health education sessions run
18 Number of patients identified
23a Number of patients assessed and treated
23b Number of patients receiving institutional-based rehabilitation services (minor surgery) and number of patients linked for further institutional rehabilitation
27 Number of patients trained in self-care

Community level

12 Improved KAP post-training compared to pre-training; HEWs satisfied with training materials; community-level intervention packages implemented
14 Number of WASH supplies delivered to patients
16 Increased % of people with severe disability who have access to rehabilitation services
17a Number of CC meetings; positive change in KAP in community
17b Patient self-help groups and/or peer support groups established
17c/d Availability of information leaflets in health posts; number of leaflets distributed; positive change in KAP in community
17e Coping skills acquisition / counselling in place; self-stigma reduced
19 Number of meetings held; number of stakeholders engaged; all relevant stakeholder groups engaged
20 Number of supervision sessions
21 Number of referrals by HEWs to health facilities
22 Number of patients self-identified
24 Number of referrals by HEWs to health facilities
25 Number of patients screened for mental health
26 HEWs satisfied with training materials; increased number of referrals to health post; positive change in KAP in community
31 Patient/families satisfied with home visit; retention in care / missed appointments; appropriate referrals
